# Underlying mechanisms of novel cuproptosis-related dihydrolipoamide branched-chain transacylase E2 (DBT) signature in sunitinib-resistant clear-cell renal cell carcinoma

**DOI:** 10.18632/aging.205504

**Published:** 2024-02-01

**Authors:** Shiue-Wei Lai, Pei-Wei Weng, Vijesh Kumar Yadav, Narpati Wesa Pikatan, Chi-Tai Yeh, Ming-Shou Hsieh, Chu-Lin Chou

**Affiliations:** 1 Division of Hematology/Oncology, Department of Internal Medicine, Tri-service General Hospital, National Defense Medical Center, Taipei, Taiwan; 2 Department of Orthopaedics, School of Medicine, College of Medicine, Taipei Medical University, Taipei, Taiwan; 3 Department of Orthopaedics, Shuang Ho Hospital, Taipei Medical University, New Taipei City, Taiwan; 4 Graduate Institute of Biomedical Materials and Tissue Engineering, College of Biomedical Engineering, Taipei Medical University, Taipei, Taiwan; 5 Department of Medical Research, Taipei Medical University Shuang-Ho Hospital, Taipei, Taiwan; 6 Continuing Education Program of Food Biotechnology Applications, College of Science and Engineering, National Taitung University, Taitung, Taiwan; 7 Department of Dentistry, Taipei Medical University-Shuang Ho Hospital, New Taipei City, Taiwan; 8 School of Dentistry, College of Oral Medicine, Taipei Medical University, Taipei City, Taiwan; 9 Division of Nephrology, Department of Internal Medicine, School of Medicine, College of Medicine, Taipei Medical University, Taipei, Taiwan; 10 Taipei Medical University-Research Center of Urology and Kidney, Taipei Medical University, Taipei, Taiwan; 11 Division of Nephrology, Department of Internal Medicine, Shuang Ho Hospital, Taipei Medical University, New Taipei, Taiwan; 12 Division of Nephrology, Department of Internal Medicine, Hsin Kuo Min Hospital, Taipei Medical University, Taoyuan City, Taiwan

**Keywords:** renal cell carcinomas, sunitinib, cuproptosis, drug resistance

## Abstract

Renal cell carcinoma (RCC) is the predominant form of malignant kidney cancer. Sunitinib, a primary treatment for advanced, inoperable, recurrent, or metastatic RCC, has shown effectiveness in some patients but is increasingly limited by drug resistance. Recently identified cuproptosis, a copper-ion-dependent form of programmed cell death, holds promise in combating cancer, particularly drug-resistant types. However, its effectiveness in treating drug resistant RCC remains to be determined. Exploring cuproptosis's regulatory mechanisms could enhance RCC treatment strategies. Our analysis of data from the GEO and TCGA databases showed that the cuproptosis-related gene DBT is markedly under expressed in RCC tissues, correlating with worse prognosis and disease progression. In our study, we investigated copper CRGs in ccRCC, noting substantial expression differences, particularly in advanced-stage tumors. We established a connection between CRG expression levels and patient survival, positioning CRGs as potential therapeutic targets for ccRCC. In drug resistant RCC cases, we found distinct expression patterns for DBT and GLS CRGs, linked to treatment resistance. Our experiments demonstrated that increasing DBT expression significantly reduces RCC cell growth and spread, underscoring its potential as a therapeutic target. This research sheds new light on the role of CRGs in ccRCC and their impact on drug resistance.

## INTRODUCTION

Renal cell carcinoma (RCC) is the ninth most common primary renal malignancy globally and has a high mortality rate. RCC occurs more frequently in individuals with obesity or high blood pressure and individuals who smoke, and its incidence rate in men is twice as high as that in women [1, 2]. Several reports have noted that RCC has multiple subtypes, among which clear-cell RCC (ccRCC) is the most common, followed by papillary RCC (pRCC), chromophobe RCC (chRCC) and other rare subtypes [3, 4]. Patients generally do not show symptoms of RCC until it progresses to an advanced stage. The disease eventually reaches a locally advanced or metastatic stage in approximately one-third of patients, and tumor recurrence and distant metastasis are common after nephrectomy [5]. Although much progress has been made in treatment in recent years, treatment options remain limited and relatively ineffective. The 5-year survival rate for people with end-stage RCC is approximately 10% [6]. The recurrence and mortality rates of RCC are more than 40%—the highest rates among the 10 most common cancers in men and women—and approximately 25% of patients with RCC have distant metastasis [7]. The most common age of RCC onset is 60–70 years, and the incidence rate of RCC in men is 1.5–2 times that in women [8]. Obesity, hypertension, smoking, and chronic kidney disease (CKD) are also risk factors for RCC [9]. The 5-year survival for end-stage RCC is approximately 10% [10]. Approximately 30% of patients with RCC experience relapse after undergoing primary tumor resection [11]. Biomarkers of RCC, such as VHL, TP53/p53, KRAS, AKT, XIAP, MCL-1, TGase 2, MDM2, HIF1α, NRF2, and HIF2α, have been identified as key indicators for the diagnosis, prognosis, and treatment of RCC [12]. Despite the discovery of such indicators and the increased detection rate of RCC during clinical examinations owing to modern tools such as ultrasonography (US) and computed tomography, the incidence of RCC continues to rise. Most cases of early-stage RCC are detected by accident, and effective treatments for patients with advanced RCC have yet to be developed [13]. Many patients with RCC are only diagnosed after the tumor has metastasized, which poses a considerable challenge for effective treatment. Therefore, identifying diagnostic indicators and formulating effective treatments are necessary for predicting and managing advanced or recurrent RCC. Cuproptosis is a novel form of cell death dependent on copper ions, has been identified as a significant factor in preventing the onset and advancement of cancer, demonstrating notable effectiveness against drug-resistant tumors. However, its efficacy in treating drug-resistant renal cell carcinoma (RCC) is still uncertain. Thus, understanding the regulatory mechanisms of cuproptosis could yield valuable insights for enhancing RCC treatment strategies. In this research, we investigated the metabolic processes involving copper and the signaling pathways related to copper, focusing on identifying potential targets associated with DBT in the context of copper-induced apoptosis.

## RESULTS

### Expression of cuproptosis-related genes (CRGs) in ccRCC cohorts

**Figure 1:**
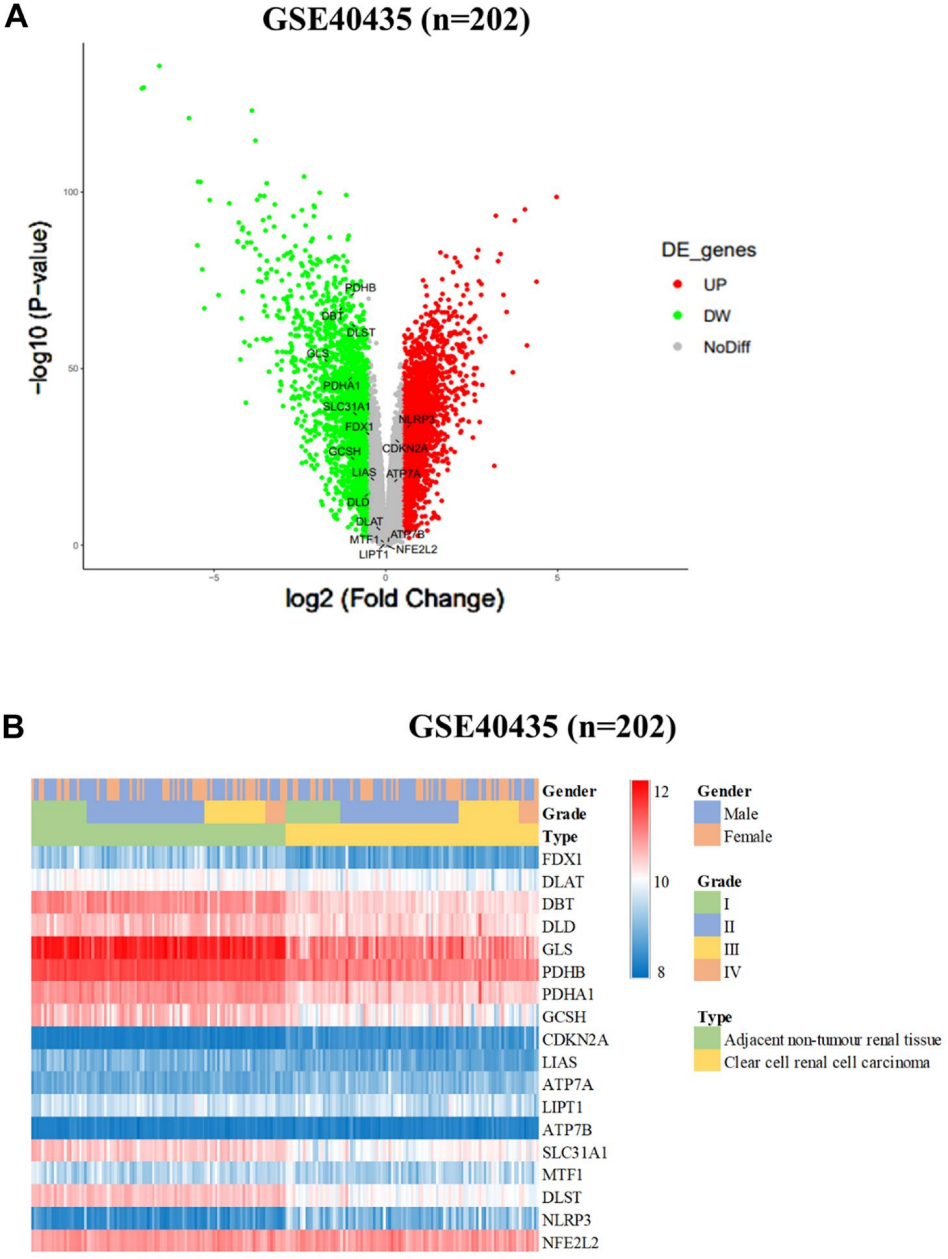
**Differential expression of cuproptosis-related genes (CRGs) in ccRCC cohorts.** (**A**) Heatmap and (**B**) Volcano plot of the differential expression of CRGs in normal tissues in ccRCC cohort GSE40435 (n = 202).

In our comprehensive study involving patients with renal cell carcinoma (RCC), we conducted an in-depth assessment of the roles played by dihydrolipoamide branched chain transacylase E2 (DBT) and genes associated with cuproptosis. These were evaluated as potential markers for predicting the prognosis of RCC. Specifically, our research aimed to investigate the hypothesis that the presence and activity of DBT and cuproptosis-related genes might be correlated with improved survival outcomes for individuals afflicted with RCC. To further this investigation, our analytical approach involved a detailed examination of various factors that could potentially affect recurrence-free survival among the RCC patients. This included a meticulous categorization of these factors based on their associated risk levels. By stratifying these factors, we aimed to create distinct risk profiles that could aid in better understanding the prognostic implications for patients. Following the stratification, our study extended to a comparative analysis of overall survival rates among the different risk groups. This comparison was crucial in highlighting the potential impact of DBT and cuproptosis-related gene expression on the overall prognosis and survival outcomes of RCC patients. By juxtaposing these survival rates across the variously classified risk groups, we sought to draw meaningful conclusions about the prognostic significance of these genetic factors in RCC. Our goal is to investigate the use of these factors as biomarkers for targeted therapy. Table 1 displays the pertinent clinical data regarding DBT expression and its corresponding correlation analysis. Figure 1 in our report presents the initial findings of our study. We conducted an *in-silico* analysis of the GSE40435 database, which contains data from 202 clear cell renal cell carcinoma (ccRCC) patients. Our analysis revealed that various cuproptosis-related genes (CRGs) exhibited altered expression levels in individuals with ccRCC. Specifically, we noticed a pattern of overexpression and downregulation of these genes in the ccRCC samples compared to normal tissues. Notably, the NLRP3 gene was found to be significantly upregulated in ccRCC tissues. This gene's elevated expression could be an important marker or a contributing factor in the disease process of ccRCC. In contrast, a group of other critical genes, namely PDHB, DBT, DLST, GLS, PDHA1, SLC31A1, FDX1, GCSH, and DLD, showed a significant reduction in their expression levels in ccRCC tissues when compared with normal kidney tissues. These genes are known to play vital roles in various cellular processes, and their downregulation in ccRCC might be indicative of the altered metabolic and regulatory pathways in this cancer type. The findings summarized in Figure 1 provide valuable insights into the gene expression patterns associated with ccRCC, particularly concerning the behavior of cuproptosis-related genes. Understanding these patterns could be crucial for developing targeted therapies and better understanding the pathophysiology of ccRCC. The figure serves as a visual representation and summary of these significant alterations in gene expression, highlighting the potential roles these genes may play in the development and progression of clear cell renal cell carcinoma.

**Table 1:** Correlation between DBT expression and clinicopathological variables of RCC patients (n=30).

**Characteristics**	**Drug therapy response**		
	**Non-recurrence (15)**	**Sunitinib-resistance (15)**	***P*-value**
Age			
• < 65 yo	10 (66%)	9 (60%)	*0.70*
• ≥ 65 yo	5 (33%)	6 (40%)	
Sex			
• Male	12 (80%)	13 (86.6%)	*0.62*
• Female	3 (20%)	2 (13.3%)	
Stage			
• Non-Metastatic	10(66.6%)	4 (26.6%)	*0.02*
• Metastatic Disease	5(33.3%)	11 (73.3%)	
Histological Grade			
• Poor	3 (20%)	13 (86.6%)	*0.00025*
• Well/Moderate	12 (80%)	2 (13.3%)	
DBT expression			
• Low	2 (13.3%)	14 (93.3%)	*0.00001*
• High	13 (86.6%)	1 (6.6%)	
SERPINE1 expression			
• Low	12 (80%)	5 (33.3%)	*0.009*
• High	3 (20%)	10 (66.6%)	
PDHB expression			
• Low	9 (60%)	13 (86.6%)	*0.098*
• High	6 (40%)	2 (13.3%)	

### Gene expression of CRGs between normal tissues and tumors by stage and sex in ccRCC

**Figure 2:**
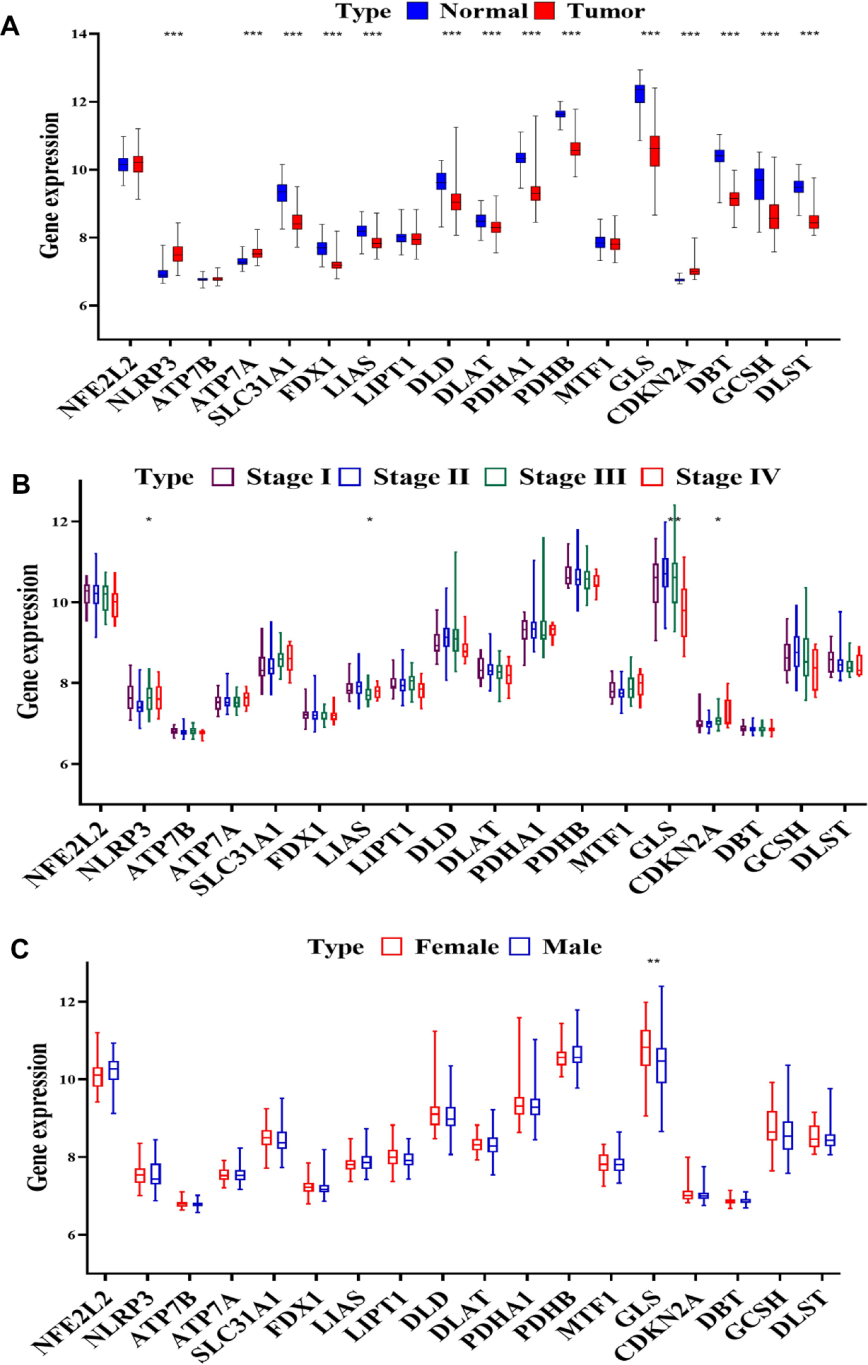
**CRGs expression in progression of ccRCC.** (**A**) Expression of CRGs in normal tissues and tumors samples from GSE40435 by (**B**) stage and (**C**) sex.

In our comprehensive study, we utilized data from the clear cell renal cell carcinoma (ccRCC) database GSE40435, which includes information from 202 patients. Our objective was to analyze and compare the expression patterns of cuproptosis-related genes (CRGs) in normal kidney tissues versus ccRCC tissues across various disease stages and between male and female patients. This analysis is visually summarized in Figure 2 of our report. Our findings revealed a complex pattern of gene expression changes in ccRCC. We observed both overexpression and downregulation of CRGs in these cancer tissues. Specifically, the NLRP3 and ATP7A genes showed overexpression, while CDKN2A was notably upregulated. In contrast, a significant downregulation was seen in several key genes, including SLC31A1, FDX1, LIAS, DLD, DLAT, PDHA1, PDHB, GLS, DBT, GCSH, and DLST (P < 0.001, as shown in Figure 2A). Furthermore, we explored the correlation between gene expression and the stage of ccRCC. Our analysis indicated that the expression of NLRP3 and CDKN2A genes positively correlated with the advancement of ccRCC stages (P < 0.05). On the other hand, LIAS and GLS gene expression showed a negative correlation with the disease stage (P < 0.05 and P < 0.01, respectively, as depicted in Figure 2B). Intriguingly, we also discovered a significant difference in GLS gene expression between male and female patients with ccRCC (P < 0.01, illustrated in Figure 2C). The patterns of gene expression observed in our study suggest that CRGs play a crucial role in the development and progression of ccRCC. The significant alterations in the expression of these genes, particularly their correlations with cancer stage and variations between sexes, underscore their potential as targets for therapeutic intervention and as biomarkers for the diagnosis and prognosis of ccRCC. These findings pave the way for further research into the molecular mechanisms of ccRCC and the development of targeted treatments based on the modulation of CRGs.

### Expression of CRGs in TCGA-KIRC cohort

**Figure 3:**
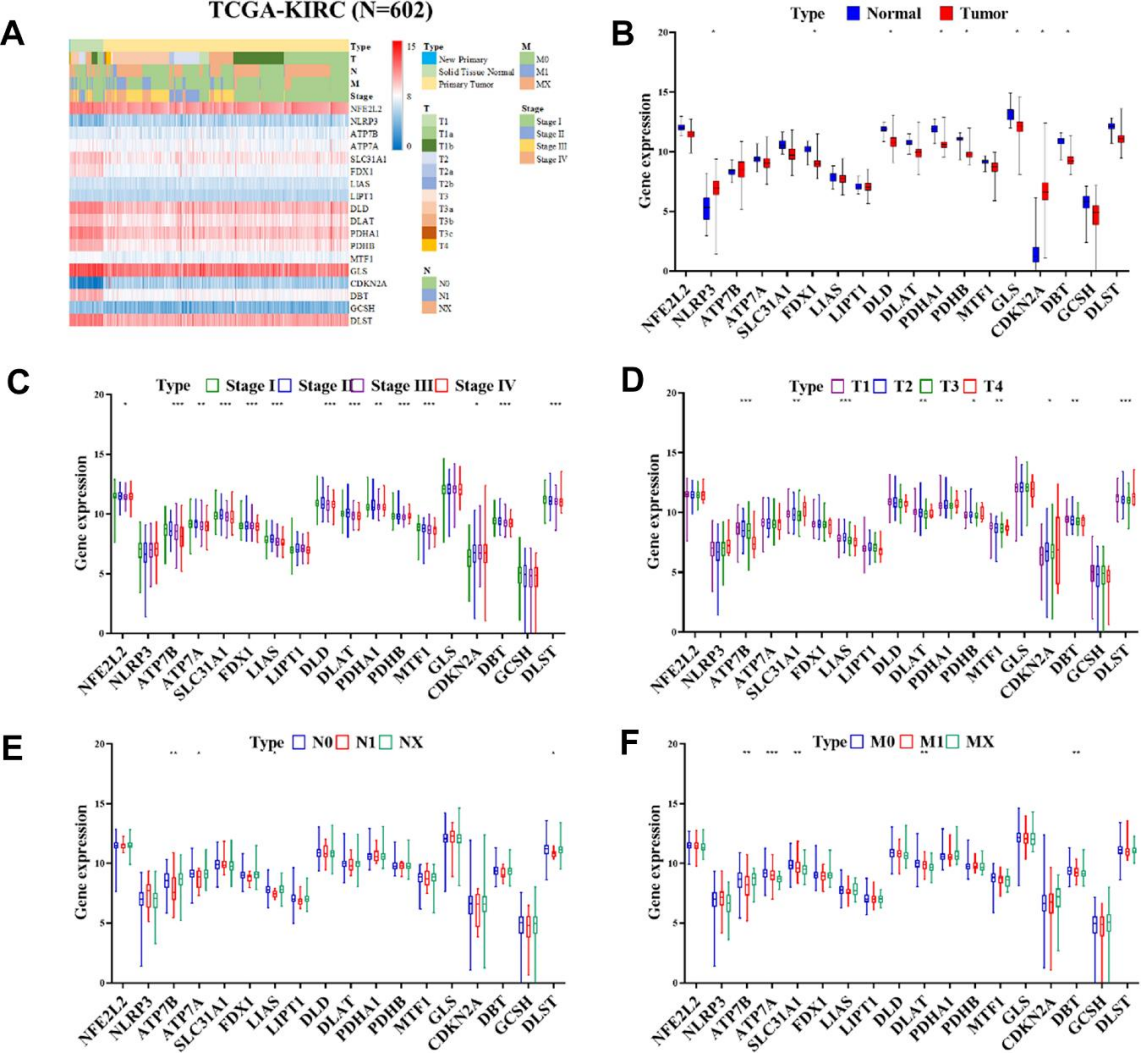
**Heatmap displaying the differential expression of CRGs expression in progression of ccRCC.** (**A**) Heatmap displaying the differential expression of CRGs in normal tissues in TCGA-KIRC cohort (n = 602). (**B**) Expression of CRGs in normal tissues and tumors samples from the TCGA-KIRC cohort by (**C**) stage, (**D**) Tumor, (**E**) Node, and (**F**) Metastasis.

Our study extended to an *in-silico* analysis of the TCGA-KIRC cohort, which is a part of the publicly accessible renal cell carcinoma (RCC) database. This analysis was focused on investigating the differential expression of cuproptosis-related genes (CRGs) in normal kidney tissues compared to RCC tissues. The results of this investigation are presented in Figure 3A, 3B. In the context of RCC tissues, we observed significant upregulation in the NLRP3 and CDKN2A genes (P < 0.05). Conversely, a group of genes, namely FDX1, DLD, PDHA1, PDHB, GLS, and DBT, exhibited significant downregulation. These findings highlight the distinct expression patterns of CRGs in RCC, suggesting their potential role in the disease's pathology. Additionally, we compared the expression of CRGs in normal tissues against tumor tissues at various stages and across different TNM stages, as illustrated in Figure 3. Our analysis within the TCGA-KIRC cohort revealed that CRG expression varied significantly. Notably, CDKN2A gene expression positively correlated with the stage of RCC. In contrast, genes such as NFE2L2, ATP7B, ATP7A, SLC31A1, FDX1, LIAS, DLD, DLAT, PDHA1, PDHB, MTF1, DBT, and DLST were found to have a negative correlation with tumor stage (as shown in Figure 3C). Moreover, our study examined the impact of CRG expression on tumor size. We found that the expression of genes including ATP7B, SLC31A1, LIAS, DLAT, PDHB, MTF1, CDKN2A, DBT, and DLST had a significant influence on the size of the tumor, as depicted in Figure 3D. Furthermore, the correlation between CRG expression and lymph node metastasis was also investigated. The expression of ATP7B, ATP7A, LIAS, and DLST showed a significant correlation with lymph node metastasis, as illustrated in Figure 3E. Lastly, we explored the relationship between CRG expression and the extent of metastasis. Here, we found that the expression levels of ATP7B, ATP7A, SLC31A1, DLAT, and DBT were significantly correlated with the degree of metastasis (Figure 3F). Overall, our in-depth analysis of the TCGA-KIRC cohort underscores the significance of CRGs in RCC, particularly in terms of their association with tumor stage, size, lymph node metastasis, and overall metastasis. These findings provide valuable insights into the molecular underpinnings of RCC and emphasize the potential of CRGs as biomarkers and therapeutic targets in this disease.

### Correlation of CRG expression with overall survival or disease-free survival in TCGA-KIRC cohort

**Figure 4:**
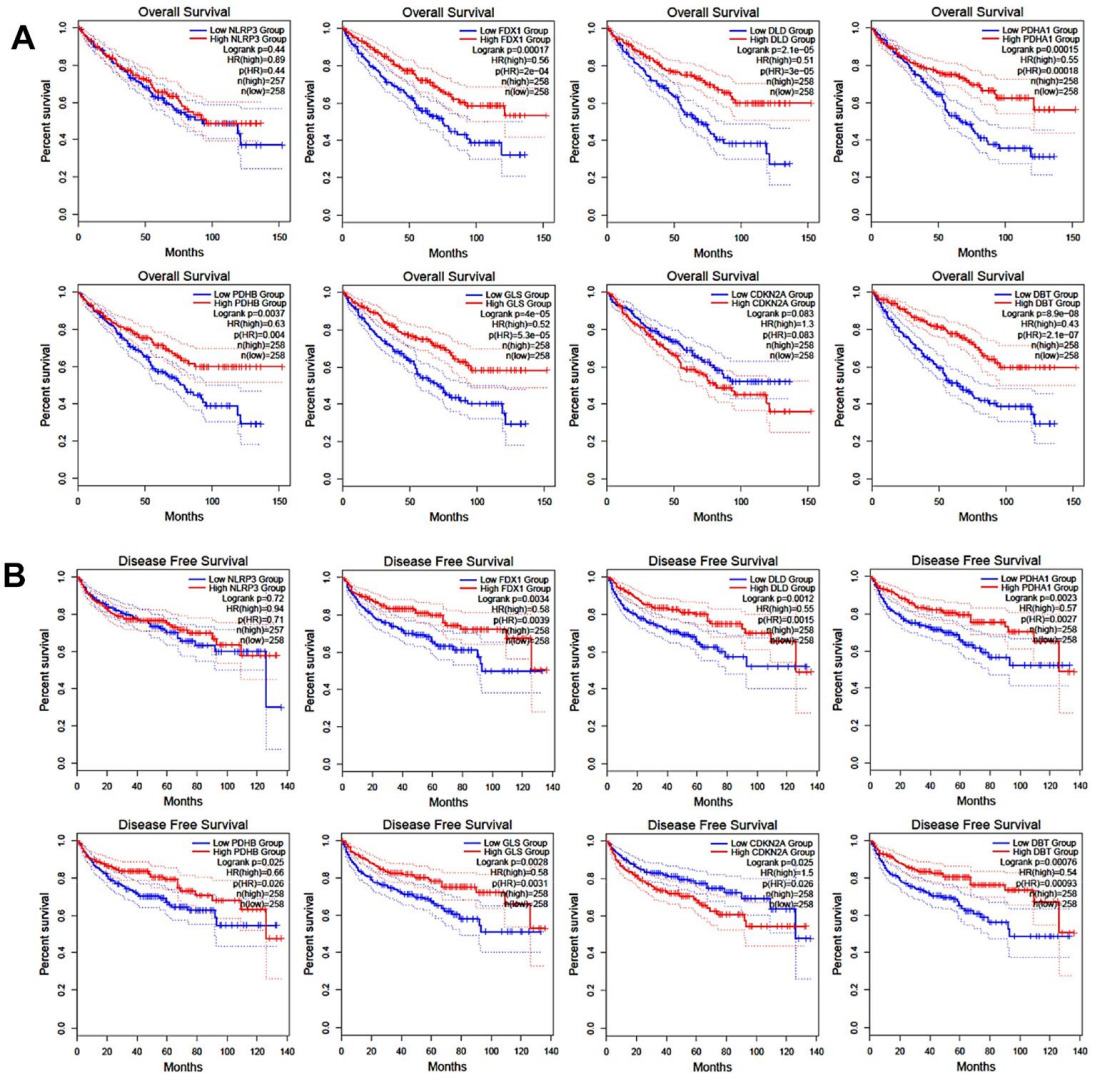
Correlation of CRGs expression with overall survival (**A**) and disease-free survival (**B**).

The *in-silico* analysis of the TCGA-KIRC (The Cancer Genome Atlas - Kidney Renal Clear Cell Carcinoma) cohort reveals a significant association between certain cancer-related genes (CRGs) and the outcomes of patients with renal cell carcinoma (RCC). Specifically, this study focused on genes such as FDX1, DLD, PDHA1, PDHB, GLS, and DBT. The analysis demonstrated that patients with RCC who have lower expression levels of these CRGs tend to experience worse overall survival (OS) outcomes compared to those with higher CRG expression levels. This correlation was statistically significant, as indicated by a *P*-value of less than 0.05, as shown in Figure 4A. Furthermore, the study also investigated the impact of CRG expression levels on disease-free survival (DFS), which is the period after treatment during which no disease is detected. It was found that patients with lower expression of certain CRGs, namely FDX1, DLD, PDHA1, PDHB, GLS, CDKN2A, and DBT, had significantly worse DFS outcomes. This finding was also statistically significant (P < 0.05), as illustrated in Figure 4B. This evidence suggests that the expression levels of these specific genes could potentially be used as biomarkers to predict survival outcomes in RCC patients. The results underscore the importance of CRGs in the pathogenesis and progression of RCC and provide valuable insights for potential therapeutic strategies and prognosis prediction.

### KEGG pathway analysis and correlation between DBT and related genes

**Figure 5:**
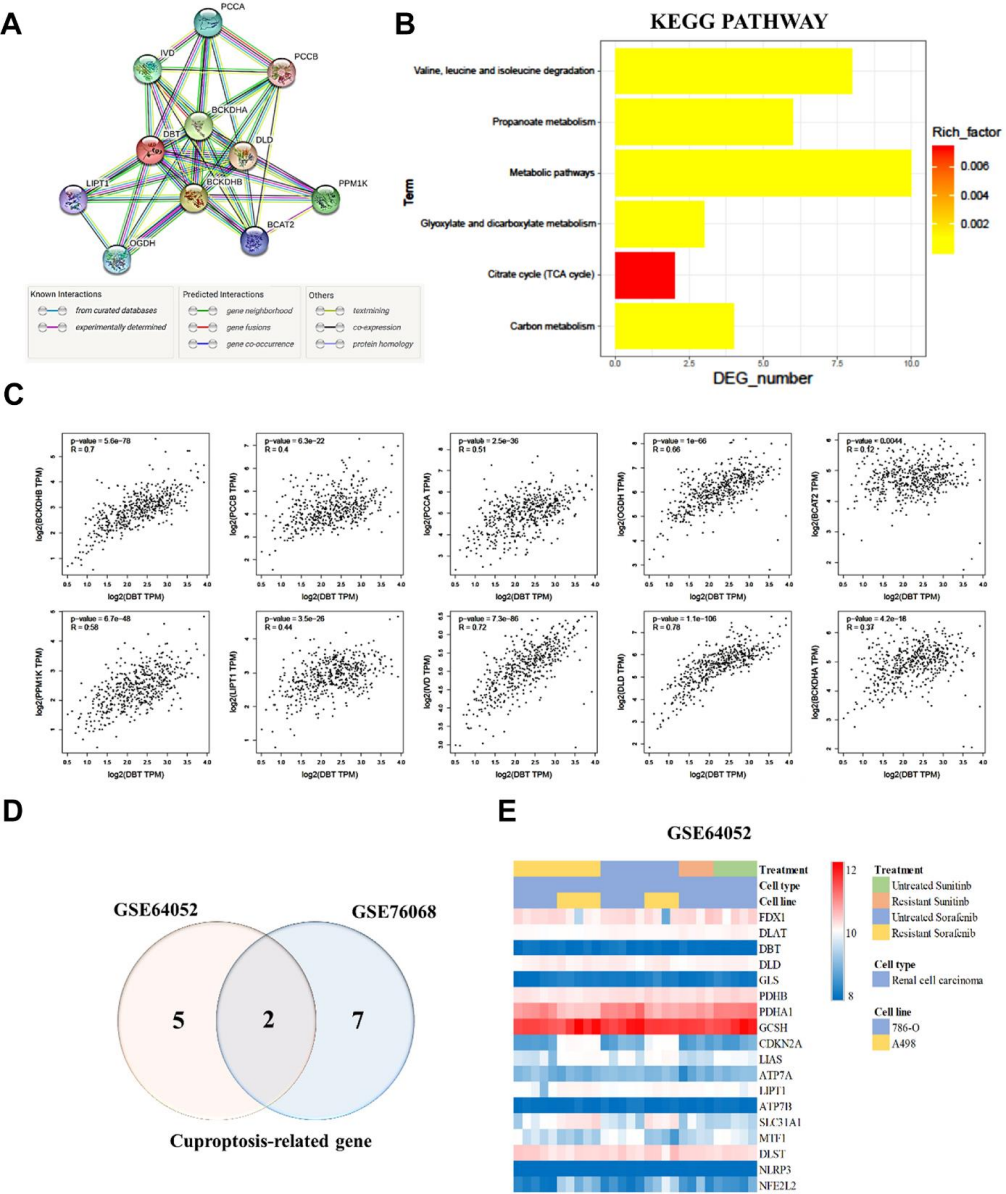
**Kyoto Encyclopedia of Genes and Genomes (KEGG) pathway analysis and correlation between DBT and related genes.** (**A**) STRING protein–protein association networks of DBT. (**B**) KEGG pathway analysis of DBT. (**C**) Correlation of DBT and related genes. (**D**) Differential expression of copper apoptosis-related genes (CRGs) in a cohort of resistant RCC. (**E**) Different colors represent data from different datasets. Overlapping regions correspond to shared differentially expressed CRGs.

To further understand the biological functions and pathways involving DBT (Dihydrolipoamide branched chain transacylase E2), an in-depth analysis was conducted using the Kyoto Encyclopedia of Genes and Genomes (KEGG) pathway analysis through the STRING database. This approach was designed to map DBT's interactions and pathways, providing insights into its role in cellular processes. The analysis identified ten DBT-related genes, which were found to be primarily enriched in several key metabolic pathways. These included the propanoate metabolism pathways, which are crucial in the breakdown of certain fatty acids and amino acids; the valine, leucine, and isoleucine degradation pathways, which are essential in the metabolism of branched-chain amino acids; the glyoxylate and dicarboxylate metabolism pathways; and the citrate cycle (TCA cycle) pathways, a central metabolic pathway critical for energy production in cells. Additionally, these genes were also involved in broader carbon metabolism and other metabolic pathways. These findings, presented in Figure 5A, 5B, underscore the diverse and critical roles of DBT in cellular metabolism. Further analysis revealed strong correlations between DBT, and other significant genes involved in these metabolic pathways. For instance, there was a notable correlation between DBT and BCKDHB (r = 0.7, P = 5.6e-78), indicating a strong positive relationship. Similar strong correlations were observed with PCCB (r = 0.4, P = 6.3e-22), PCCA (r = 0.51, P = 2.5e-36), and OGDH (r = 0.66, P = 1e-66). Other correlations, though less strong, were still statistically significant, such as with BCAT2 (r = 0.12, P = 0.0044), PPM1K (r = 0.58, P = 6.7e-48), LIPT1 (r = 0.44, P = 3.5e-26), IVD (r = 0.72, P = 7.3e-86), DLD (r = 0.78, P = 1.1e-106), and BCKDHA (r = 0.37, P = 4.2e-18), as shown in Figure 5C. The *in-silico* analysis of two distinct publicly available datasets of drug-resistant renal cell carcinoma (RCC) cohorts, specifically GSE64052 and GSE76068, highlighted a noteworthy association between certain cancer-related genes (CRGs) and resistance in RCC. The findings, illustrated in Figure 5D, was based on a comprehensive analysis of these datasets, aiming to understand the genetic underpinnings of drug resistance in RCC. Contains standard correlations with cuproptosis-related genes (Figure 5E). The study involved an in-depth examination of the differential expression of CRGs in drug resistant RCC by utilizing these two independent cohorts. This approach was crucial in identifying the specific CRGs that played a role in RCC resistance. Understanding the roles of DBT and GLS in RCC drug resistance can provide valuable insights into developing new therapeutic strategies aimed at overcoming or preventing drug resistance in RCC. This research paves the way for future studies to further explore the implications of these genes in RCC and possibly in other types of cancer where drug resistance is a major challenge. These correlations suggest a complex network of interactions where DBT and these related genes contribute to various metabolic processes. The strong statistical significance of these correlations (*P*-values) indicates a high likelihood that these relationships are not due to chance, providing robust evidence for the involvement of DBT in these essential metabolic pathways. Understanding these interactions and pathways is crucial for comprehending the role of DBT in normal cellular function and its potential implications in diseases, including cancer.

### Mechanisms identified for DBT-regulated genes involved in RCC aggressiveness

**Figure 6:**
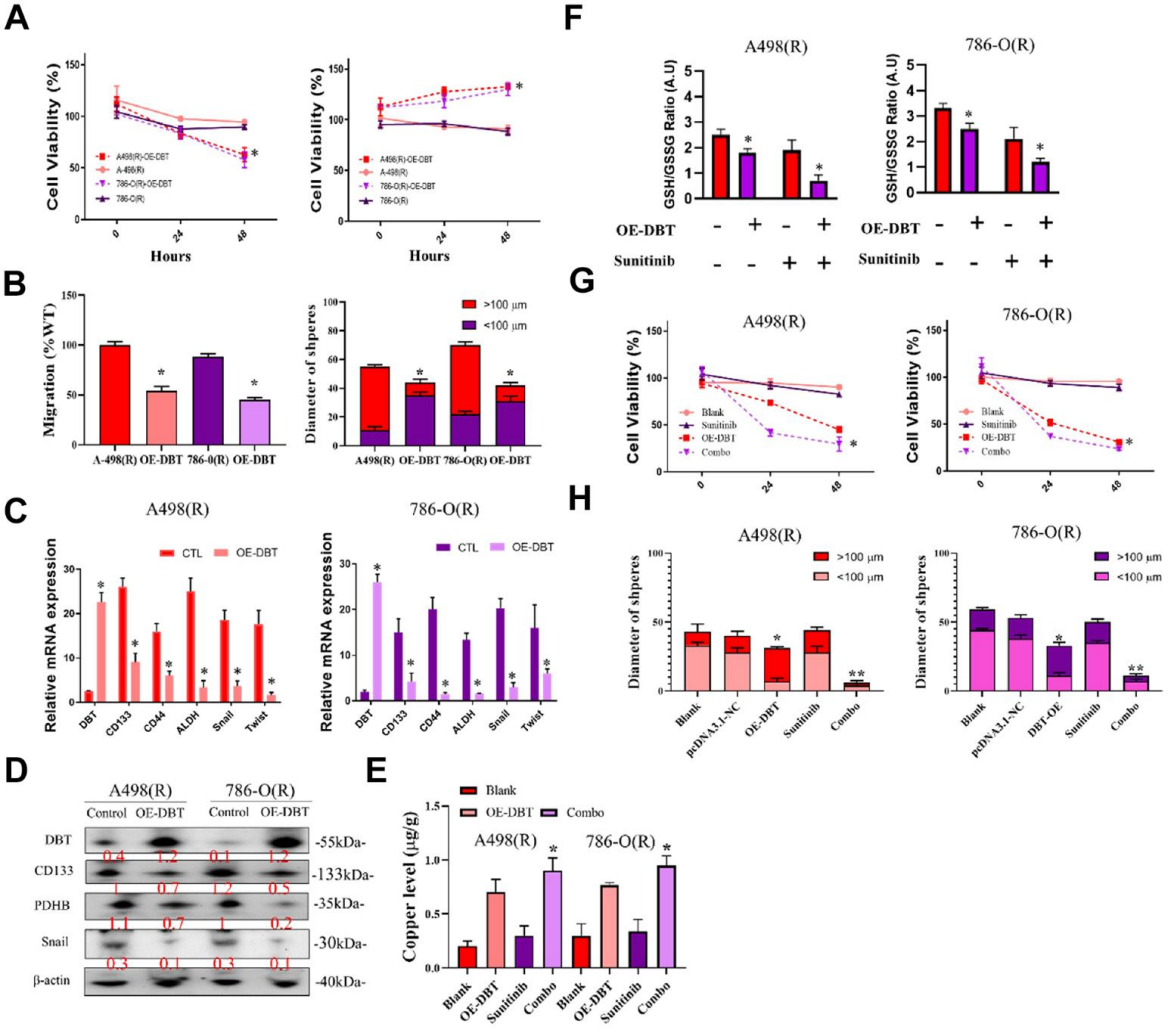
**Analysis of DBT overexpression in sunitinib-resistant RCC cell Lines.** (**A**) Cell Viability Assessment: Comparative analysis of cell viability in sunitinib-resistant RCC cell lines, 786(R)-O and A498(R) following transfection with OE-DBT and control plasmid (pCDNA3.1). (**B**) Wound Healing and Tumor Sphere Formation Assays: Post-24-hour scraping, this figure illustrates the reduced migration rate in DBT-overexpressing cells compared to control RCC cells. Additionally, a decrease in tumor sphere formation diameter is highlighted. (**C**) RT-qPCR Analysis: Validation of transfection efficiency and assessment of gene expression changes related to copper apoptosis and cell function in DBT-overexpressed RCC cell lines. (**D**) Western Blot Analysis: Confirmation of successful transfection and evaluation of protein expression alterations corresponding to cellular functions in DBT-overexpressed cells. (**E**) Intracellular Copper Ion Levels: Depiction of copper ion concentrations within the cells, emphasizing the effect of DBT overexpression. (**F**) GSH/GSSG Ratio Assay. (**G**, **H**) Combination Treatment Efficacy: Demonstrating the impact of combination treatments on re-sensitizing drug-resistant cells. This includes reductions in both tumor sphere formation diameter and cell viability in DBT-overexpressed RCC cell lines. (*p < 0.05, **p < 0.01).

We first manipulated DBT-KD or DBT overexpression and examined the subsequent transcriptome changes to discover novel effectors in DBT-regulated gene axes. RNA from cells with DBT overexpression or DBT knockdown was extracted and subjected to microarray analysis. Transcripts that exhibited similar changes in both groups were selected for subsequent functional assessment. Ingenuity pathway analysis and gene ontology analysis was subsequently performed to functionally classify the identified genes, enabling the researchers to identify potential genes that play a role in cell motility, extracellular matrix adhesion, or metastasis. Real-time RT-PCR was then performed in DBT-manipulated cells to validate the changes observed during microarray analysis. For genes known to be involved in regulating cell migration or metastasis, reporter constructs were generated and analyzed to determine DBT dependence. The ultimate goal of this process was to narrow down the candidate genes to a small set of key genes for subsequent evaluation. To delve deeper into the biological functions of DBT in kidney cancer, specifically in kidney renal clear cell carcinoma (KRCC), we conducted a detailed examination of DBT's mRNA and protein levels in two renal cell carcinoma (RCC) cell lines: 786-O and A498. This analysis utilized RT-qPCR and Western blotting techniques. Given the relatively low expression of DBT, we employed overexpression plasmid (OE-DBT) and a control plasmid (pCDNA3.1) for transfection into Sunitinib-resistant 786(R)-O and A498(R) cell lines, thereby amplifying DBT expression. Our findings, as depicted in Figure 6A, revealed the impact of this overexpression on the cell viability in these sunitinib-resistant RCC cell lines. To further assess the functional implications of DBT overexpression, we executed wound healing assays and tumor sphere formation tests. The results, observed 24 hours post-scraping, indicated a marked reduction in the migration rate of the DBT-overexpressing (OE-DBT) cells compared to control RCC cells. Additionally, the OE-DBT cells demonstrated a decreased capability in forming tumor spheres, as shown by the reduced diameter of these spheres (Figure 6B). We validated the efficiency of the transfection and the consequent alterations in gene regulation related to copper apoptosis and cell function through both RT-qPCR (Figure 6C) and Western blot (Figure 6D). The intracellular levels of copper ions, crucial for understanding the underlying mechanisms, are illustrated in Figure 6E. GSH acts as a thiol-based chelator of copper, inhibiting cuproptosis. Being a natural chelator of intracellular copper ions, a reduction in GSH levels corresponds with a rise in intracellular copper concentration. This is illustrated in the GSH/GSSG Ratio Assay presented in Figure 6F. Furthermore, our experiments suggest that combining treatments can restore sensitivity in drug-resistant cells while simultaneously reducing both the diameter of tumor sphere formation and overall cell viability (Figure 6G, 6H). This comprehensive approach sheds light on the significant role DBT plays in the cellular processes of RCC, highlighting its potential as a therapeutic target in the treatment of RCC.

## DISCUSSION

Renal eradication surgery, the main treatment for RCC, has achieved promising results because it can effectively remove primary tumors [14]. However, this procedure causes loss of kidney function, which can lead to other adverse effects, such as the emergence of CKD [2]. Patients with stage I–III RCC can undergo partial resection or nephrectomy, but relapse occurs in 33% of these patients. Chemotherapy, which is commonly used to treat other malignant tumors, is ineffective for treating RCC and is thus not recommended [14]. Conversely, patients with end-stage local RCC or metastatic RCC are treated with systemic therapy, including immune checkpoint inhibitors such as nivolumab and pembrolizumab, which target PD-1; avelumab and atezolizumab, which target PD-L1; ipilimumab, which targets CTLA-4; axitinib, sunitinib, pazopanib, and bevacizumab, which target vascular endothelial growth factor (VEGF); and the mTOR inhibitor everolimus [15]. Tyrosine kinase inhibitors (TKIs), mTOR inhibitors, and VEGF inhibitors are molecularly targeted drugs frequently used to treat RCC and have been demonstrated to improve the overall prognosis of patients with unresectable or metastatic RCC. TKIs can inhibit or even eliminate tumor growth and spread and are thus frequently used to treat multiple types of cancer [2]. Sunitinib, a TKI, is the first targeted drug for metastatic RCC treatment that has been approved by the FDA. Sunitinib and pazopanib, another TKI, are viable first-line treatments for RCC [5]. In addition to TKIs, anti-VEGF monoclonal antibodies have been demonstrated to be useful for managing advanced RCC [6]. As the development of targeted drugs has increased treatment options in recent years and these drugs are in widespread use in clinical applications, resistance to targeted drugs has also emerged and has limited their effectiveness. Therefore, identifying the mechanism of drug resistance and corresponding treatment methods is crucial.

Copper is an essential micronutrient for humans and is a key catalytic cofactor involved in many biological processes such as mitochondrial function, synthesis of biological compounds, and antioxidation. Normal physiological and biochemical processes require the copper concentration in the body to be strictly regulated and maintained within a specific range. Copper levels are balanced through several biochemical processes, including copper absorption, intracellular utilization, distribution, and excretion [16]. In adults, the tissue concentrations of copper are approximately 4–6 μg/g in the liver, 7–12 μg/g in the kidney, 3–5 μg/g in the brain, 1 μg/g in muscle, and 1000 ng/ml in plasma and serum [17]. Dietary copper is absorbed mainly in the intestines, especially the duodenum. In this process, copper transporter 1 (CTR1) absorbs copper into enterocytes, where Cu ^2+^ is subsequently reduced by the metalloreductase activity of six-transmembrane prostate epithelial antigen (STEAP), duodenal cytochrome b (DCYTB) 6, and DCYTB 7. The resulting Cu ^+^ is absorbed by intestinal cells, converted into Cu ^2+^, and released into the blood, where it is bound to ceruloplasmin, albumin, copper chaperone proteins, histidine, and macroglobulin in the blood and transported to various tissues and organs [18–20]. The ATP7A protein is responsible for transporting copper into the cells of tissues and organs. However, mutations in this protein that inhibit its ability to transfer copper cause copper-related genetic diseases or Menkes disease (copper deficiency), leading to copper imbalance, difficulty eating, seizures, muscle atrophy, weakness in the hands and feet, reduced ankle reflexes, delayed neurodevelopment, and other problems. These symptoms usually manifest in infants when they are 6–12 weeks of age [21]. Wilson disease (excess copper) is caused by different mutations of the ATP7B gene. When the ATP7B protein, which is responsible for excreting copper from the liver, is mutated, copper accumulates in tissues throughout the body, primarily in the brain and the liver, causing lipid peroxidation and mitochondrial dysfunction [22].

Copper plays a role in various molecular pathways in cancer that exert proliferative or proangiogenic effects on tumors, causing them to grow and spread [23]. Many copper-binding proteins associated with cancer have been identified, such as antioxidant-1 (Atox1), which is involved in the cell cycle, cell proliferation, angiogenesis, and vascular remodeling [24]. Moreover, lung cancer patients who have higher ATP7A expression exhibit poorer responses to cisplatin-based chemotherapy [25], and ATP7B and ATP7A are associated with resistance to cisplatin-based drugs in breast and ovarian cancer [26]. Ctr1, another copper-binding protein, is also involved in the transport of platinum-based antineoplastic drugs [27]. These findings demonstrate the key role of copper-associated proteins in cancer, their involvement in cancer progression, and the development of resistance to cisplatin-based chemotherapy drugs. Copper has also been reported to be involved in the mitogen-activated protein kinase (MAPLK) pathway and to regulate cancer cell apoptosis, survival, cell differentiation, cell proliferation, and metastasis [28].

Cuprotosis differs from well-known apoptosis and ferroptosis. Cuprotosis is a newly discovered regulated cell death that is triggered by Cu ^2+^, and it occurs when copper accumulates in cells and causes noncaspase-, apoptosis-, or reactive oxygen species (ROS)-induced cell death [29]. Copper plays an essential role in the regulation of cell proliferation, angiogenesis, and metastasis [30], and it is also involved in ROS generation [31]. Excess copper leads to proteotoxic stress and induces apoptosis through protein lipidation, mitochondrial respiration, lipidated protein aggregation, and Fe–S cluster protein destabilization [32]. Enhanced mitochondrial metabolism has been reported in melanoma, breast cancer, and leukemia, and drug-resistant cancer cells rely on the energy obtained through mitochondrial respiration [33]. Mitochondrial respiration regulates cancer proliferation, migration, and growth [34]. Cuprotosis involves copper binding to the fatty acylation group in the citric acid (TCA) cycle, and during mitochondrial respiration, the increase in fatty acylated TCA enzymes leads to the abnormal aggregation of fatty acylated proteins and the dysfunction of Fe–S-containing proteins, causing acute proteotoxic effects that trigger cell death [30]. In melanoma, liver cancer, and breast cancer, copper chelators can limit the bioavailability of copper and inhibit cancer progression and the metabolic process underlying cancer cell survival [31]. Cu ^2+^ is transported to the mitochondria through ionophores, is reduced to Cu ^1+^ by ferredoxin (Fdx1) and combines with dihydrolipoyllysine-residue acetyltransferase (DLAT), a lipidation component in the TCA cycle. This leads to lipidated protein aggregation and the instability of Fe–S cluster proteins, resulting in proteotoxic oxidative stress that triggers cell death. Moreover, a key advantage of this program is that it is not inhibited by other well-known cell death inhibitors [30].

Branched-chain amino acids (BCAAs), such as isoleucine, leucine, and valine, are essential amino acids created by plants, bacteria, and fungi. Animals cannot synthesize BCAAs and consequently must obtain BCAAs from their diet [35, 36]. BCAAs are catabolized in the mitochondria and cytoplasm of cells, where they are first converted into branched-chain α-keto acids, 2-ketoisocaproate, 2-keto-3-methylvalerate, and 2-ketoisovalerate. Oxidative decarboxylation subsequently transforms branched-chain α-ketoacid dehydrogenase (BCKD) into branched-chain acyl-CoA esters, CO_2_, and NADH. BCKD has three components: heterotetrameric (α2β2), decarboxylase E1, and dihydrolipoamide. Branched-chain transacylase E2 and FAD-dependent dihydrolipoyl dehydrogenase E3 are closely related to BCKD and play similar roles [37]. In non–small-cell lung cancer (NSCLC), BCAAs are used as nitrogen sources, and the dysfunction of the enzymes branched-chain amino acid transaminase (BCAT) 1 and BCAT2, which are responsible for BCAA usage, impairs NSCLC tumor formation [38]. Glioblastoma cells highly express BCAT1, which initiates the catabolism of BCAAs. Inhibiting BCAT1 can subsequently repress tumor growth, proliferation, and invasion [39]. Inhibiting BCAT1 also elevates αKG levels, impairing acute myeloid leukemia (AML) stem cell function [40]. In breast cancer, knockdown of BCAT1 eliminates BCAA catabolism, which inhibits mTOR-mediated mitochondrial biogenesis and function, repressing cancer proliferation [41]. Dysregulation of BCAA metabolism activates mTORC1, promoting the development and progression of hepatocellular carcinoma catabolism [42]. BCAA regulates the activity of mTOR, and BCAA metabolism is believed to contribute to cancer progression and may also participate in multiple pathways of carcinogenesis [43]. Therefore, the role of BCAA metabolism in cancer warrants further exploration.

As mentioned in the earlier text, copper ions and cuproptosis play a key role in cancer proliferation and metastasis, which suggests that copper can be used in cancer treatments. Therefore, copper chelators and copper ionophores are in high demand as targeted cancer drugs. Many copper ionophore drugs have been used to treat cancer, including 8-hydroxyquinolines (HQs), flavones, dithiocarbamates, and bis(thiosemicarbazone) ligands [44]. Although the concept of cuproptosis had not yet been proposed when copper ionophores were first developed, their tumor suppressor effects are well-documented. Disulfiram, an FDA-approved treatment for chronic alcoholism, has been demonstrated to have differential anticancer effects in numerous solid tumors and hematological malignancies [45]. The bis (thiosemicarbazonato) copper complexes glyoxalbis N4-methylthiosemicarbazonato]Cu(II) [Cu(II)(gtsm)] and diacetylbis[N4-methylthiosemicarbazonato]Cu(II) [Cu(II)(atsm)] have been demonstrated to inhibit proteasome chymotrypsin-like activity that targets prostate cancer cells [46]. Elesclomol has been verified as an effective treatment for metastatic melanoma, leukemia, and various solid tumors [47], because it inhibits cancer cell growth by inducing oxidative stress and copper uptake in cells [48]. Elesclomol sensitivity is positively correlated with the degree of dependence of cancer cells on mitochondrial metabolism [47]. Disulfiram (DSF) is a drug approved by the US FDA for the treatment of alcoholism, but studies have demonstrated that DSF can be used for the treatment of melanoma by inhibiting the ubiquitin-proteasome system, reactive oxygen species, and death signaling pathways [49]. Clioquinol (CQ) has been widely used to treat skin infections, and the functionalization of CQ, namely the 8-HQ scaffold, confers substantial anticancer properties. This activity is enhanced by Cu, and CQ inhibits the growth of cancer cells through the inhibition of the proteasome, cell motility and metastasis, and generation of oxidative stress [50]. Thiosemiccarbazones (TSCs), which are chelating ligands, can be divided into single and double TSCs, with an example being 3-aminopyridine carboxaldehyde (3-AP, Triapine). TSC can chelate iron to interfere with tumor cell metabolism and proliferation [51]. Copper chelators and copper ionophores are potential cancer treatments because they can cause copper depletion or overload, perturbing copper homeostasis in tumors.

Prior research has indicated that in various human cancers, the expression of DBT (dihydrolipoamide branched chain transacylase E2) is often reduced. Specifically, in kidney renal clear cell carcinoma (KIRC), lower DBT levels are linked to more severe clinicopathological characteristics and a worse prognosis for patients. According to both univariate and multivariate Cox regression analyses, DBT can serve as an independent predictor of outcomes in KIRC patients. Experimentally, increasing DBT expression in KIRC cells through plasmid introduction has been shown to slow their growth and decrease their ability to migrate and invade. Further analysis suggests that DBT might play a role in pathways related to immunotherapy and drug metabolism. The CIBERSORT algorithm analysis implies that DBT may enhance anti-cancer immune responses in KIRC by activating M1 macrophages, mast cells, and dendritic cells, and concurrently reducing regulatory T cell activity. Lastly, DBT expression in KIRC has been found to have a strong association with immune checkpoints and the efficacy of targeted and immunotherapy drugs [52].

In our comprehensive analysis, we focused on the expression patterns of copper CRGs within a specific ccRCC cohort. Our findings revealed a significant variation in the expression of CRGs in ccRCC tumors, with this differential expression becoming more pronounced in tumors that were at an advanced stage (as depicted in Figure 1). This notable disparity in CRG expression was further corroborated by our observations in the TCGA-KIRC cohort. Here, we noticed a strong association between elevated levels of CRG expression and more advanced tumor stages, as well as higher TNM stages (illustrated in Figure 2).

Our research also included an in-depth examination of the relationship between CRG expression and patient outcomes, specifically overall survival (OS) and disease-free survival (DFS). The results of this analysis indicated a potential link between CRG expression levels and these crucial clinical outcomes, suggesting that targeting CRGs might offer a novel and effective therapeutic strategy for ccRCC. This hypothesis, however, requires further investigation, as emphasized in Figures 3, 4. We extended our research to include an analysis of CRG expression in two distinct drug resistant RCC cohorts. In this phase of our study, we discovered that two specific CRGs—DBT and GLS—exhibited significantly different expression profiles in drug-resistant RCC tumors. Notably, these differences in expression were strongly correlated with the tumors’ resistance to treatment (as shown in Figure 5). This finding adds a new dimension to our understanding of drug resistance in RCC.

In addition to these observational studies, we conducted experimental work to explore the functional implications of these findings. We observed that overexpression of DBT in RCC cells led to a noticeable decrease in cell growth, migration, and invasion. These experimental outcomes, detailed in Figure 6, underscore the potential of DBT as a target for therapeutic intervention in RCC, particularly in the context of its role in influencing tumor behavior and treatment resistance. This comprehensive approach, integrating both observational and experimental data, provides a more nuanced understanding of the role of CRGs in ccRCC and opens new avenues for research and treatment strategies.

## CONCLUSIONS

**Figure 7:**
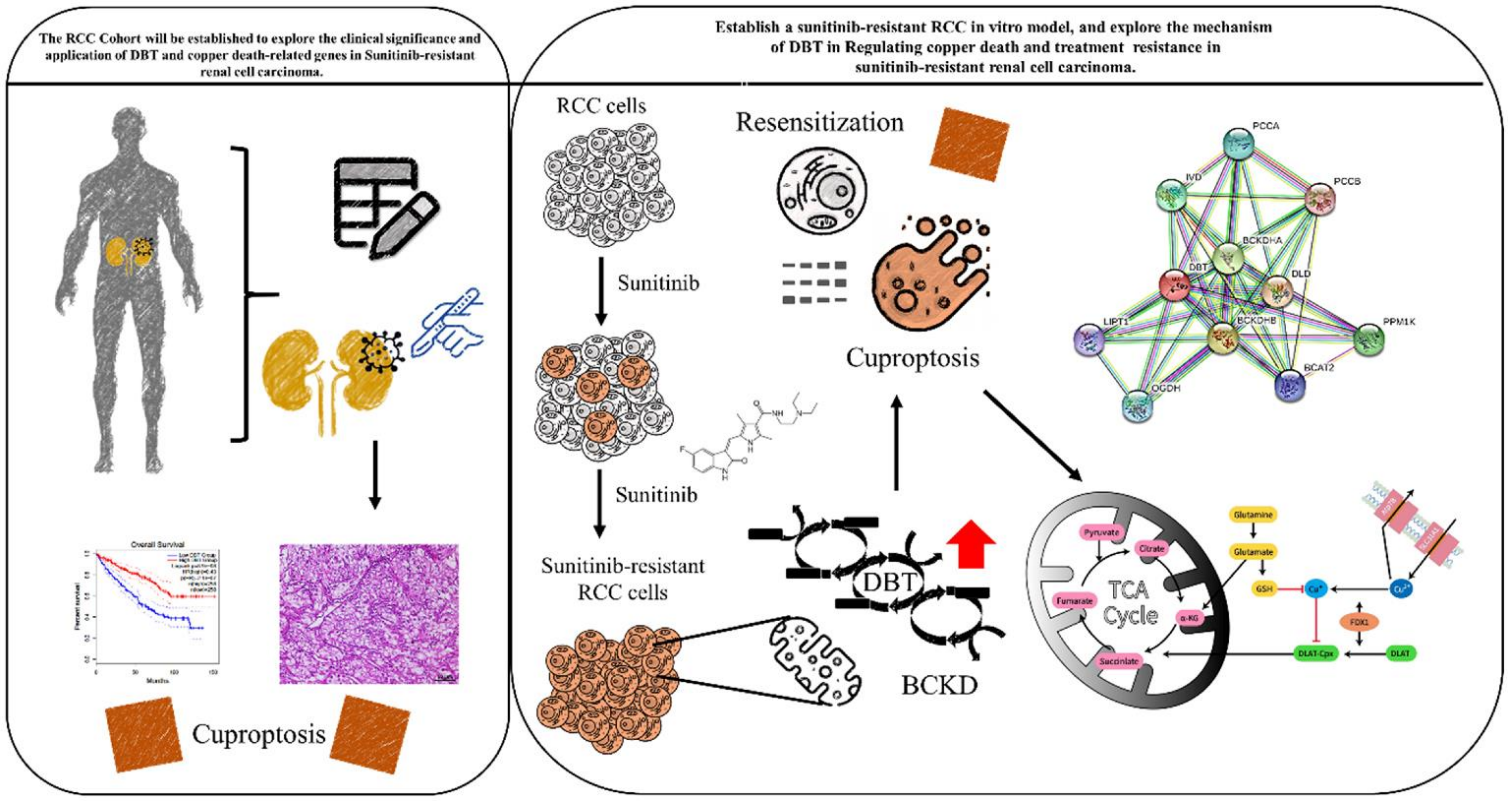
Graphic abstract for executing this research project will provide insight into how cuproptosis affects the development of RCC and the molecular mechanism of drug resistance, and hopefully helps to develop novel therapeutic strategies to treat drug-resistant renal cell carcinoma.

Figure 7 presents a graphical abstract illustrating the research project's execution. This project aims to shed light on the impact of cuproptosis in the progression of Renal Cell Carcinoma (RCC) and to unravel the molecular mechanisms underlying drug resistance. The ultimate goal of this study is to pave the way for the development of innovative therapeutic approaches for the treatment of drug resistant RCC. This study aims to determine the significance of dihydrolipoamide branched chain transacylase E2 (DBT) in patients with Sunitinib-Resistant Clear-Cell Renal Cell Carcinoma (RCC). We found that DBT expression is reduced in various cancers, and in RCC, low DBT levels are associated with advanced disease characteristics and poor prognosis. The study examined DBT's predictive value in various cancers, highlighting its significant prognostic importance in RCC, leading to further investigations. Through comprehensive bioinformatics analysis, we explored the relationship between DBT and clinicopathological features in RCC patients. Our findings indicate a significant reduction in DBT expression in RCC, correlating with disease progression and lower survival rates. Experimental results showed that DBT overexpression inhibited RCC cell growth, migration, and invasion. Moreover, we uncovered DBT's role in the tumor immune environment, suggesting that DBT may contribute to cancer development by initiating abnormal inflammatory and immune reactions. This research enhances our understanding of RCC pathophysiology and potential molecular targets.

## MATERIALS AND METHODS

### Clinical significance of DBT- and cuproptosis-related genes in the development of drug-resistant RCC

The research focused on examining the expression of dihydrolipoamide branched-chain transacylase E2 (DBT) and genes related to cuproptosis in drug-resistant renal cell carcinoma (RCC) patients. This was done by analyzing tissue samples from these patients, collected at TMU-SHH Hospital, through immunohistochemistry. The study adhered to the guidelines of the Declaration of Helsinki and received approval from the Institutional Review Board of SHH. Participants provided informed consent, and their tissue samples, collected between January 2016 and July 2021, were retrospectively analyzed. Patients underwent comprehensive pre-treatment evaluations including clinical history, physical exams, and various diagnostic tests such as barium swallow X-ray, blood chemistry, CT, MRI, angiography, abdominal ultrasound, PET, and kidney biopsy. Treatment followed SHH protocols and NCCN guidelines. The study assessed the expression levels of DBT- and cuproptosis-related genes and their correlation with clinical factors like sex, age, tumor stage, lymphocyte infiltration, vascular invasion, and TNM stage. Patient follow-up was regular, and the impact of these genes on survival rates, response duration, and recurrence timing was investigated using multivariate and Kaplan–Meier analyses. Additionally, the study used data from The Cancer Genome Atlas (TCGA) and the Gene Expression Omnibus (GEO) databases to analyze the expression of these genes in drug-resistant RCC. For this study, RNA sequencing data originally in FPKM format was transformed into TPM format. We maintained both clinical and RNA sequencing data, ensuring a thorough evaluation in accordance with the publishing guidelines of The Cancer Genome Atlas (TCGA). Since the data utilized in this research was sourced from public databases like TCGA and the Gene Expression Omnibus (GEO), the requirement for informed consent and ethical approval was deemed unnecessary. Furthermore, this study was conducted in compliance with all relevant publishing standards of the aforementioned databases. The Expression Project for Oncology (expO) was also utilized, integrating gene expression data with clinical annotations to enhance understanding of human cancers and aid in identifying diagnostic and prognostic markers, as well as therapeutic targets.

### *In vitro* cell culture functional studies

The study explored the impact of DBT on the growth and survival of cells using two approaches: enhancing DBT function (via ectopic expression in renal cell carcinoma, or RCC, cell lines) and reducing DBT function (through knockdown or knockout using shRNA in RCC cell lines). Cell viability was measured using the SRB assay. The A-498 cell line (HTB-44™, ATCC), derived from a 52-year-old female kidney cancer patient, has epithelial characteristics, and is effective for transfection, making it useful in cancer research. The 786-O cell line (CRL-1932, ATCC), with epithelial-like features, was sourced in 1975 from a 63-year-old White female with renal cell adenocarcinoma. A498 cell lines were cultured in MEM medium, while 769-P cell lines were cultured in RPMI1640 medium (Gibco, NY, USA). Medium with a recommended 10% fetal bovine serum (FBS), at a temperature of 37° C and 5% CO_2_ atmosphere. The growth medium was refreshed every 48 to 72 hours. Anchorage-independent cell growth (assayed by soft agar colony formation) was used to evaluate the effect of DBT on cell migration and invasion. The construction of both the control overexpression plasmid (referred to as Vector) and the DBT overexpression plasmid (labeled as OE-DBT) was accomplished using the pcDNA 3.1(+) vector as a base. To introduce these plasmids into A498 cells, a transfection process was employed, utilizing Lipofectamine 3000 transfection reagent, a product of Invitrogen, located in Waltham, Massachusetts, USA. This procedure was carried out strictly adhering to the guidelines provided by the manufacturer. shRNAs targeting identified DBT-regulated genes were purchased from the RNAi Core Facility of Academia Sinica, a research academy in Taiwan. Successful knockdown cells verified by quantitative RT-PCR or Western blotting were established. Transwell migration and invasion assays were performed on these cells to investigate whether they are involved in regulating cell migration. Morphological changes including cell shape and cytoskeletal rearrangements were also examined. A key segment of the functional assessment involved knocking out well-known cell motility regulators or validated genes in DBT-manipulated cells to evaluate whether they can rescue the migratory or invasive ability of DBT. Total RNA was extracted from both tissues and cells using the Trizol method. Subsequently, cDNA synthesis was conducted using the TaKaRa kit (RR047A), which was then applied to real-time quantitative PCR (qPCR) using the TAKARA kit (RR420A). The 2^−ΔΔCt^ method was employed for data analysis. Specific primers were used in the real-time PCR process Supplementary Table 1.

### Development and identification of sunitinib resistance in ccRCC cell models

To create sunitinib-resistant (R) variants of human ccRCC cell lines, we consistently exposed A498 and 786-O cells to escalating doses of sunitinib. The cells were deemed stably resistant when they showed marked insensitivity to a high dose (10 µM) of the drug, accompanied by noticeable accumulation of sunitinib within the cells, particularly in the lysosomes. The unique auto-fluorescence properties of sunitinib (excitation: 420 ± 20 nm, emission >470 nm) enabled us to visualize its presence in the cells without additional staining or altering the compound. This resistance was established over 30 weeks of bi-weekly treatments during cell passaging, as evidenced by an increased effective dose required to reduce cell viability by 50% (ED50). The cells were continually exposed to 1 µM sunitinib at each cell passage until they were used in experiments.

### Molecular mechanisms of DBT-triggered hallmarks of carcinogenesis

We examined the involvement of known components of the DBT oncogenic axis to identify DBT-induced cancer hallmarks and reveal the molecular mechanisms underlying DBT-mediated functions. For each pathway, dominant negative mutants and selective pharmacological inhibitors were tested. Pathway-associated polymerase chain reaction (PCR) or functional reporter assays were performed to identify the possible involvement of other signaling molecules or DBT downstream of each pathway.

### Role of DBT in drug resistance in RCC treatments

We examined whether DBT overexpression or KD affects the drug resistance of RCC cells to a panel of chemotherapeutic agents. We conducted this procedure in a clinical setting, where resistance to therapy and relapse are strongly associated with the presence of cancer stem cell signatures. We dissected the oncogenic axis of DBT to gain insight into the signaling molecules and pathways involved in mediating drug response.

### Role of DBT in supporting cancer stemness in RCC

To characterize DBT-induced cancer stemness, we assessed the effect of DBT on the maintenance of a hierarchical pattern during RCC cell proliferation and its effect on cell self-renewal. Another goal was to determine whether DBT induces cancer stemness in RCC by sustaining the cell’s ability to generate progeny with self-limited proliferative capacity. This was determined by measuring the hepatic spheroid-forming capacity of repeating spheroids that were derived from limiting dilutions of DBT-overexpressing and depleted cells. We used the common CD133, CD44, and ALDH biomarkers to identify and characterize RCC stem cell phenotypes. We also dissected the oncogenic axis of DBT to elucidate the signaling molecules and pathways involved in supporting cancer stemness in RCC Supplementary Figure 2.

### Overexpression effect of DBT on migratory ability and proteasomal degradation in RCC

RCC cell lines were treated with DBT overexpression to compare migratory capacity through the time-lapse migration assay. Briefly, experimental cells were seeded onto 6-cm tissue culture dishes coated with collagen (10 µg/ml, 3 ml). The medium was cultured overnight and then replaced with serum-free conditioned medium. The movement of the cells was observed using an inverted microscope (Axio Observer Z1, Zeiss) with a PLAN objective 5 × (0.55 NA) magnification in a 37° C environmental chamber. Images were captured with a CCD camera (AxioCam MRm, Zeiss) at 20-min intervals for a total of 16 h and saved using MetaMorph software (Molecular Devices Corporation, Sunnyvale, CA). The cumulative distance of cell migration was determined by tracking the position of the nuclei using the trackpoint function of the NIH ImageJ software.

### Testing novel DBT overexpression in combination with chemotherapy in RCC

The antibody arrays were used to assay the activity of the cells in both the absence and presence of signaling drugs or combination treatments Supplementary Table 2. To further assess the effect of candidate molecules and DBT on cancer stemness, we determined the expression of stem cell markers in RCC tissues extracted from patients who responded to chemotherapy and those who did not respond to it and analyzed the correlations between certain stem cell markers and the chemotherapy response.

### Combination strategy testing DBT overexpression–sunitinib for drug-resistant RCC

The antibody arrays were used to assay the activity of the cells in the absence and presence of signaling drugs or under combination treatments. To further evaluate the effect of candidate molecules and the combination treatment of DBT overexpression and sunitinib against drug resistant RCC, we determined the expression of cancer stemness in tumor tissues and analyzed its correlation with tumor parameters (Supplementary Figure 1).

### Copper ion detection

The Copper (Cu) Colorimetric Assay Kit (Catalog No. E-BC-K300-M, Elabscience) operates on the principle where copper ions in the sample react with 3,5-DiBr-PAESA in acidic conditions, forming a purple complex. This complex exhibits its highest absorption peak at 580 nm. By measuring the optical density (OD) value at this wavelength, the copper ion content in the sample can be indirectly determined.

### GSH/GSSG ratio assay

Glutathione (GSH), a natural intracellular chelator of copper ions, diminishes in concentration, leading to a rise in intracellular copper levels. The GSH/GSSG Ratio Detection Assay Kit (Fluorometric - Green) from Abcam (ab138881) was utilized to measure the GSH/GSSG ratio, a critical marker of the cellular redox state. This kit, known for its accuracy and sensitivity, employs a colorimetric technique to quantify both the reduced (GSH) and oxidized (GSSG) forms of glutathione. The process involves homogenizing cells or tissues and precipitating proteins to extract total glutathione. GSSG is then selectively masked to eliminate interference in GSH measurement. Next, GSSG is converted back to GSH using a reducer, forming a chromophore detectable by colorimetric analysis. The absorbance at specific wavelengths is measured to determine the concentrations of GSH and GSSG. The ratio of GSH to GSSG is calculated, offering insights into the cellular environment's redox balance.

### Statistics

Statistical evaluations were performed using a one-way ANOVA test in GraphPad Prism 8.0.2 (San Diego, CA, USA). To assess the statistical significance of survival differences among various groups, a log-rank test was employed. A *p*-value of less than 0.05 was deemed to indicate statistical significance.

### Availability of data and materials

The Datasets that are used and analyzed by the current investigation will be provided by the corresponding author in reply to the reasonable demands.

## Supplementary Material

Supplementary Figures

Supplementary Tables
