# Supplementary material for: Underlying mechanisms of novel cuproptosis-related dihydrolipoamide branched-chain transacylase E2 (DBT) signature in sunitinib-resistant clear-cell renal cell carcinoma
Source: Aging (Albany NY). 2024 Feb 1;16(3):2679–701. doi: 10.18632/aging.205504 (PMC10911363; doi:10.18632/aging.205504)
Supplement: Supplementary Figures [file aging-16-205504-s001.pdf]

SUPPLEMENTARY FIGURES

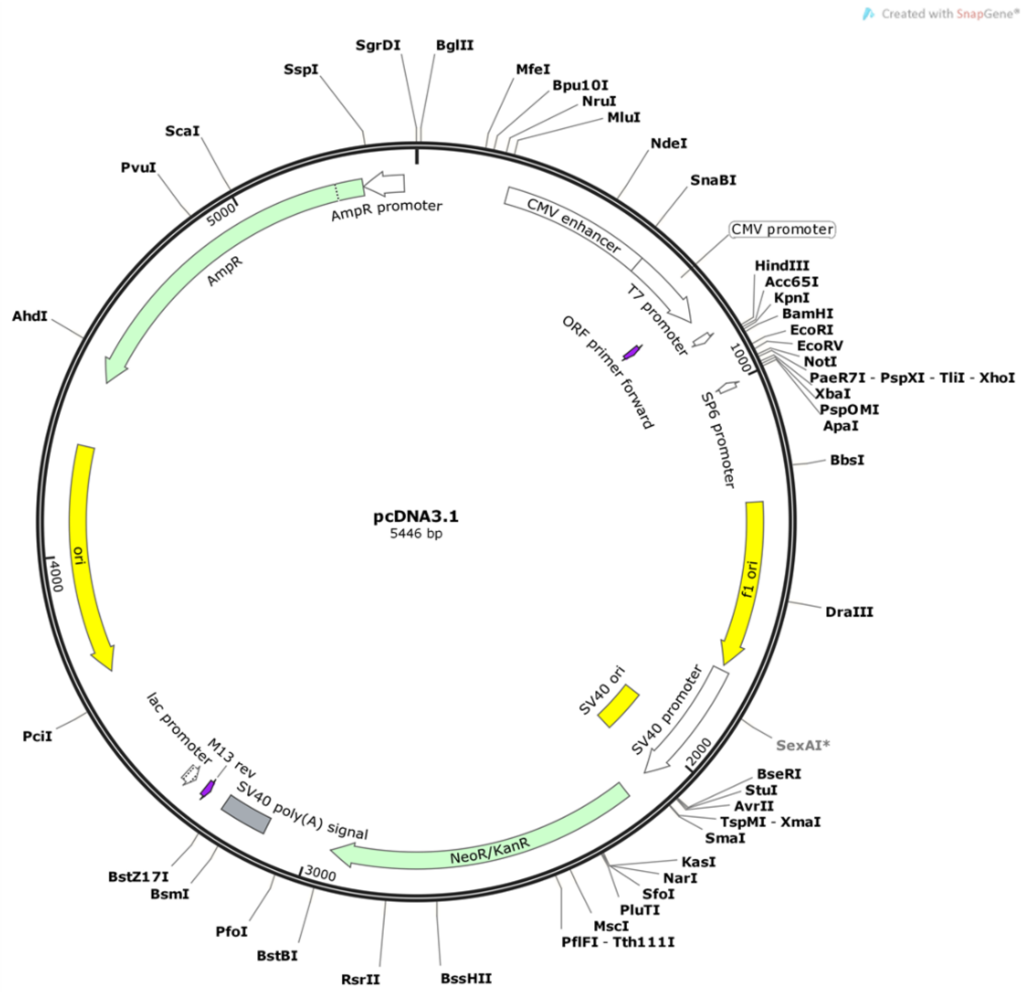

Primer	Sequence
DBT-ECOR1-forward	AATT GAATTC TCATCATGAGACACTGCAGGA
DBT-Xba1-reversed	AATT TCTAGA CCTATGTGGGGAAGCCATTA

Supplementary Figure 1. Overexpression of DBT plasmid backbone and primer design.

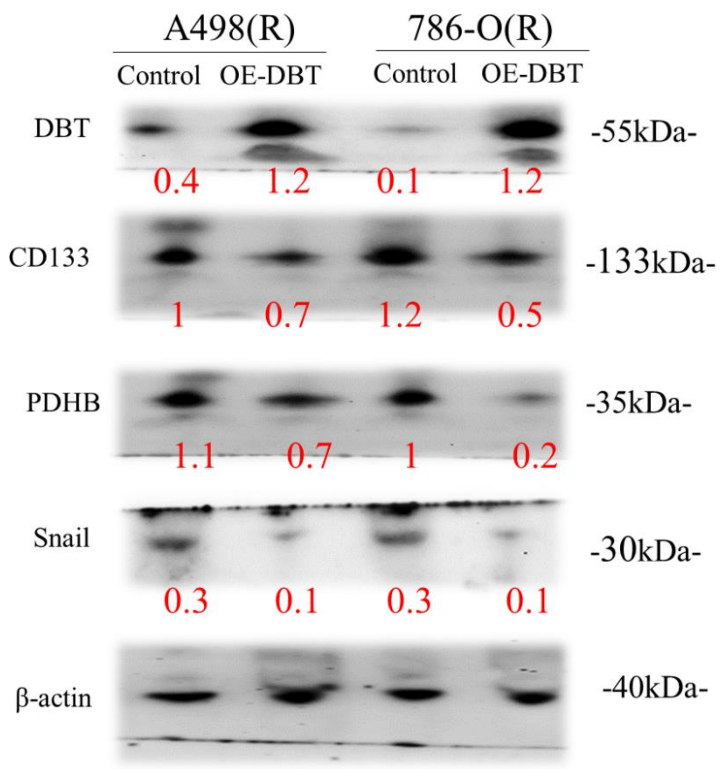

Supplementary Figure 2. Full-size blots of Figure 6D.
