# Supplementary material for: Underlying mechanisms of novel cuproptosis-related dihydrolipoamide branched-chain transacylase E2 (DBT) signature in sunitinib-resistant clear-cell renal cell carcinoma
Source: Aging (Albany NY). 2024 Feb 1;16(3):2679–701. doi: 10.18632/aging.205504 (PMC10911363; doi:10.18632/aging.205504)
Supplement: Supplementary Tables [file aging-16-205504-s002.pdf]

SUPPLEMENTARY TABLES

Supplementary Table 1. q-PCR primer list.

Primer	Forward	Reverse
DBT	TCATCATGAGACACTGCAGGA	CCTATGTGGGGAAGCCATTA
CD133	CACTACCAAGGACAAGGCGTTC	CAACGCCTCTTTGGTCTCCTTG
CD44	CCAGAAGGAACAGTGGTTTGGC	ACTGTCCTCTGGGCTTGGTGTT
Snail	TGCCCTCAAGATGCACATCCGA	GGGACAGGAGAAGGGCTTCTC
Twist	GCCAGGTACATCGACTTCCTCT	TCCATCCTCCAGACCGAGAAGG
GAPDH	GTCTCCTCTGACTTCAACAGCG	ACCACCCTGTTGCTGTAGCCAA

Supplementary Table 2. Antibody list.

Antibody	Catalog number	Source
DBT mAb	#24972	Cellsignaling
CD133 mAb	ab222782	Abcam
PDHB mAb	ab155996	Abcam
Snail mAb	ab216347	Abcam
β-Actin mAb	#4967	Cellsignaling
